# Therapeutic effect of photodynamic therapy for nonresectable cholangiocarcinoma: Protocol for a meta-analysis and systematic review

**DOI:** 10.1097/MD.0000000000009863

**Published:** 2018-02-23

**Authors:** Junjie Liu, Ping Xue, Jingwen Deng

**Affiliations:** a Department of General Surgery, Guangzhou Panyu Central Hospital; b Department of Hepatobiliary Surgery, The Second Affiliated Hospital of Guangzhou Medical University; c Central Laboratory, Guangdong Provincial Hospital of Chinese Medicine, Guangzhou, China.

**Keywords:** cholangiocarcinoma, photodynamic, protocol, systematic review

## Abstract

**Background:**

Cholangiocarcinoma is a malignant neoplasia that originates in the bile ducts. Most patients with cholangiocarcinoma are inoperable at the time of diagnosis. photodynamic therapy (PDT) is a fairly well accepted treatment in clinical practice for nonresectable cholangiocarcinoma (NCC) but lack of quantitatively assessment. Herein, we present a protocol for a systematic review to identify the efficacy of PDT in patients with NCC.

**Methods:**

We will search PUBMED, SpringerLink, Cochrane Library, the Chinese Biomedical database (CBM), WanFang data, China National Knowledge Infrastructure (CNKI) up to December 2017. Studies will be screened by title, abstract, and full text independently and in duplicate. Studies that report PDT in patients with nonresectable cholangiocarcinoma will be eligible for inclusion. Outcome variables will be assessed included survival benefit, health status and quality of life, and adverse events with photodynamic therapy. Assessment of risk of bias and data synthesis will be performed using Revman software. The hazard ratios will be extracted from the survival curves using Tierney Method. Heterogeneity among studies will be assessed using the *I*^2^ statistic.

**Results:**

This study will review randomized controlled trials, cohort studies, or retrospective studies and quantitatively assess the efficacy of PDT in patients with NCC for the latest evidence-based recommendation.

**Conclusion:**

This study will evaluate therapeutic effect of PDT in patients with NCC systematically. We expect that the results from this systematic review for clinical trials will help inform clinical practice in NCC.

## 1 Introduction

Cholangiocarcinoma is an uncommon adenocarcinoma arising from the epithelial cells of bile ducts, located along extrahepatic and intrahepatic biliary tree, excluding the ampulla of vater and the gall bladder.^[1]^ After hepatocellular carcinoma, cholangiocarcinoma is the second most common primary malignant tumor of the liver affecting 1 to 2/100,000 of the world population per year.^[2]^ However, over the past few decades, the incidence of cholangiocarcinoma has been increasing worldwide.^[3]^ Risk factors for cholangiocarcinoma include cirrhosis, primary sclerosing cholangitis, some congenital liver malformations, infection with *Opisthorchis viverrini* and *Clonorchis sinensis* and exposure to Thorotrast (thorium dioxide).^[4–11]^ However, most people with cholangiocarcinoma have no definite risk factors. In most instances, cholangiocarcinoma is difficult to diagnose at early stage, making the curative resection rather difficult.

In nonresectable cholangiocarcinoma (NCC), survival of untreated patients is only about 5 to 9 months, with effective palliative treatment can prolong the survival period.^[12]^ In addition to the best supportive treatment, endoscopic stent is a major palliative treatment for persistent drainage of the bile duct. Photodynamic therapy (PDT) is another well-known treatment for NCC.

In PDT, a nontoxic photosensitizing agent such as photofrin is given intravenously 48 hours before transpapillary or percutaneous radiation with light of a specific wave length. Due to the accumulation in the neoplastic tissue, the reactive oxygen free radicals produced by photosensitizers cause the destruction of the light absorption on the target cancer cells. The effect of PDT on survival benefit in NCC has been evaluated in numerous pioneer studies. In 1991, McCaughan reported the first experience of a woman with histologically proven adenocarcinoma of the common bile duct that was successfully treated with 6 injections of dihematoporphyrin ether followed by 7 photodynamic therapy treatments. The patient lived for 4 years after the PDT treatment.^[13]^ Since then, various controlled trials have revealed that PDT has a promising future in nonresectable cholangiocarcinoma.^[14–27]^

However, quantitatively assessment of the effect of PDT for the treatment of NCC is lacking, so as to a standard protocol of PDT for the treatment of NCC is short of foundation. Our review will evaluate therapeutic effect of PDT for the treatment of NCC systematically. We expect that the results from this systematic review for clinical trials will help inform clinical practice in NCC.

## 2 Methods and analysis

### 2.1 Eligibility criteria

We will select studies according to the eligibility criteria informed in Table 1.

**Table 1:**
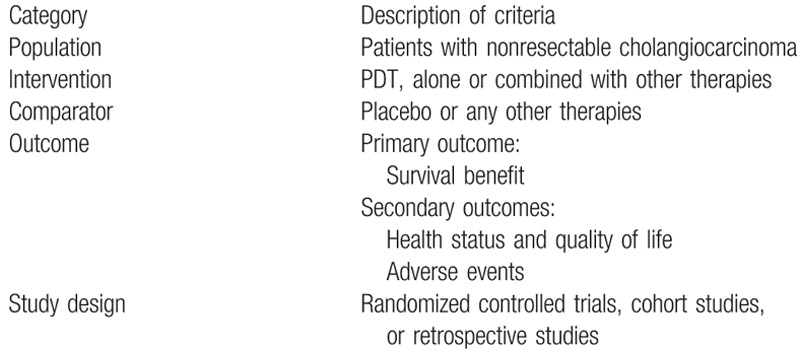
Study eligibility criteria.

### 2.2 Types of studies

Randomized controlled trials, cohort studies, or retrospective studies will be included in the systematic review irrespective of languages. We will exclude single-arm interventional studies. We will include unpublished grey literature and abstracts only if the data and methodological descriptions were stated clearly or obtained by contacting with the authors.

### 2.3 Types of participants

We will include only trials with participants whose diagnosis of NCC was established by imageology examination or pathological examination of surgical specimens. According to the definition of cholangiocarcinoma, trials with participants with cancer of gallbladder and ampulla of Vater will be excluded.

### 2.4 Types of interventions

We will include studies evaluating PDT (alone or combined with other therapies) compared with control (placebo or any other therapies).

### 2.5 Types of outcome measures

#### 2.5.1 Primary outcomes

- Survival benefit (mortality)

We will define survival of participants as the time from the start of PDT to the time of death by any reasons.

#### 2.5.2 Secondary outcomes

- Health status and quality of life

Health status and quality of life are patient-reported outcomes that describe individuals’ self-perceived health status. Questionnaires are used as measurement tools for these outcomes, for example, Karnofsky performance scoring (KPS); functional assessment of cancer therapy-hepatobiliary cancers (FACT-Hep 2015).

- Adverse events

We will define adverse events and withdrawals for any reason stated in the included studies.

### 2.6 Search strategy

We will search PUBMED, SpringerLink, Cochrane Library, the Chinese Biomedical database (CBM), WanFang data, China National Knowledge Infrastructure (CNKI) up to December 2017. Clinical trial registries will be searched to identify ongoing or recently completed trials or systematic reviews. In order to further ensure a comprehensive literature search, we will examine reference lists of included studies or relevant reviews identified through the search. We will include unpublished grey literature and abstracts only if the data and methodological descriptions were stated clearly or obtained by contacting with the authors.

The literature search strategies will be designed using keywords related to PDT as well as cholangiocarcinoma. A draft of the MEDLINE (OVID interface) search strategy for PDT and cholangiocarcinoma is shown in Table 2.

**Table 2:**
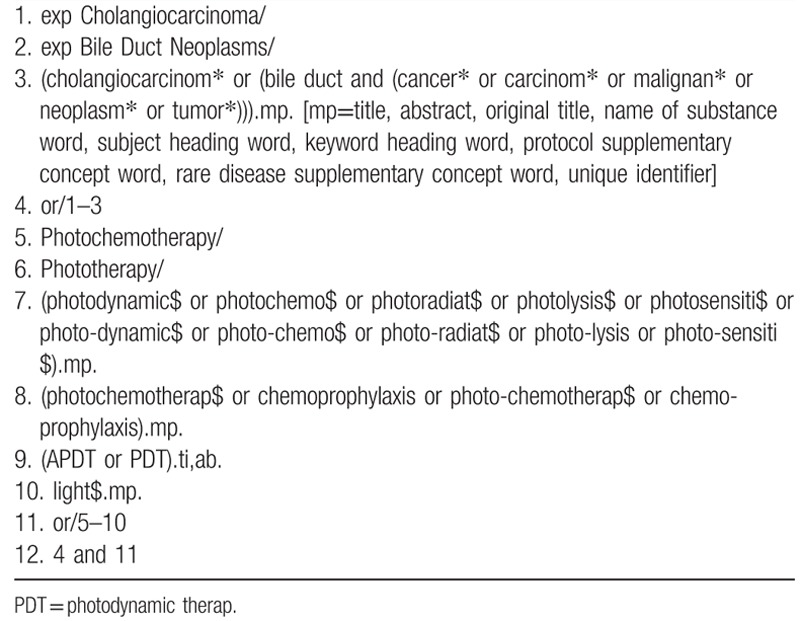
MEDLINE (OVID) search strategy.

Studies will be screened by title, abstract, and then full text (if necessary) independently by the 2 investigators. After the primary screening, the investigators will go through the full text according to the eligibility criteria. Potential disagreement during the screening process will be solved by discussion and consensus with the help of a third investigator. This process will be recorded in detail for creating a preferred reporting items for systematic reviews and meta-analysis (PRISMA) flow diagram.

### 2.7 Data extraction

All data extraction will be performed by 2 investigators by using predesigned electronic data collection forms independently. Epidata version 2.0 (Odense, Denmark) will be applied for recording, assessing, and correcting data entry errors. Information extraction will include methods, participants, interventions, outcomes, and funding sources. Since most of the publications did not report hazard ratio as outcome directly, we will extract and analyze the outcome from the Kaplan–Meier curve by Tierney method.^[28]^

### 2.8 Quality assessment

Two investigators will independently assess the quality of included studies according to the following components, as advised in the Cochrane Handbook for Systematic Reviews of Interventions^[29]^:

(a) the method of random sequence generation
(b) the method of allocation concealment
(c) the methods of blinding of participants, researchers, and outcome assessors
(d) the number of the participants lost to follow up in each arm and the reasons for losses
(e) whether all participants are analyzed according to their originally randomized group, that is, intention-to-treat (ITT) analysis
(f) whether there are other problems that can put the study at high risk of bias, like baseline imbalance, deviation from the study protocol, dropouts or withdrawals from treatment, or insensitive outcome measurement tools; and
(g) selective reporting of outcomes.

We resolved disagreements by discussion with a third investigator. We contacted the trialists to seek clarification where necessary.

### 2.9 Quality of evidence

We will evaluate the quality of evidence for the outcomes by using the Grading of Recommendations Assessment, Development and Evaluation (GRADE) system.^[30]^ The quality of evidence will be evaluated across the domains of risk of bias, consistency, directness, precision, and publication bias. According to GRADE, the quality of evidence can be rated as high, moderate, low, and very low, which is reflecting the strength of clinical recommendation.

### 2.10 Measures of treatment effect and data synthesis

We will summarize data of survival outcome and express the intervention effect by using a hazard ratio. A hazard ratio is interpreted in a similar way to a risk ratio, as it describes that if participants accept experiments rather than control intervention, they will suffer more (or less) harm at a particular time. Risk ratios (RR) and 95% confidence intervals (CI) will be calculated for dichotomous variables. We will calculate mean differences (MD) and 95% CIs for continuous outcomes using similar scales, standardized mean differences (SMD) and 95% CIs for continuous outcomes using different scales. For adverse events, we will just describe them.

Heterogeneity of effect sizes in the pooled proportions will be calculated among included studies using the Cochrane *I*^2^ statistic. If the *I*^2^ statistic is <50% with reasonable clinical homogeneity, we will conduct meta-analysis using a fixed-effect model to calculate a pooled intervention effect estimate across trials. If the *I*^2^ statistic is 50% to 80%, we will apply a random-effects model. If statistic is >80%, it means there is severe heterogeneity, we will not conduct a meta-analysis. Where it is inappropriate or impossible to perform a meta-analysis, we will summarize the data narratively for each trial.

Data will be analyzed using RevMan version 5.3.5 (The Cochrane Collaboration, The Nordic Cochrane Centre, Copenhagen. Denmark).

### 2.11 Subgroup analyses

Subgroup analysis will be performed to identify any subpopulations that may be associated with the effectiveness of PDT alone or combined with different therapy. Other factors such as different type of NCC (extrahepatic cholangiocarcinoma or intrahepatic cholangiocarcinoma) and different control interventions will be taken into account.

### 2.12 Sensitivity analysis

The robustness of the review conclusions will be verified in sensitivity analysis. And if possible, we will repeat the analysis after every low-methodological-quality study is excluded.

## 3 Discussion

PDT is generally used as an adjunctive treatment on cancer alongside surgery, chemotherapy, or radiotherapy. Ortner et al^[14]^ and Cheon et al^[15]^ reported a prolonged survival in patients with NCC who were treated with stenting plus subsequent PDT when compared with stenting alone. In Witzigmann study, PDT with stenting offered a significant survival benefit when compared with stenting alone and has a similar survival time compared with incomplete R1 and R2 resection.^[16]^ Kahaleh reported endoscopic retrograde cholangio-pancreatography (ERCP) with PDT resulted in longer survival in patients with NCC versus ERCP alone.^[17]^ In Wentrup study, PDT with a gemcitabine-based combination therapy might increase survival in patients with hilar NCC.^[18]^

Although PDT is a fairly well accepted treatment in clinical practice for NCC, it has no yet to be fully explored by quantitatively assessment. Based on above evidences, this systematic review will clarify the effectiveness of PDT on patients with NCC and identify gaps in current practice and knowledge. We expect that the results from this clinical systematic review will help inform the design of clinical trials.

### 3.1 Review status

- Preliminary searches: started.
- Piloting of the study selection process: started.
- Formal screening of search results against eligibility criteria: not started.
- Date extraction: not started.
- Risk of bias (quality) assessment: not started.
- Data analysis: not started.
